# Supplementary figures and images for: Hypothetical Pathogenetic Model of Membranous Nephropathy
Source: Int J Mol Sci. 2025 Feb 28;26(5):2206. doi: 10.3390/ijms26052206 (PMC11900195; doi:10.3390/ijms26052206)

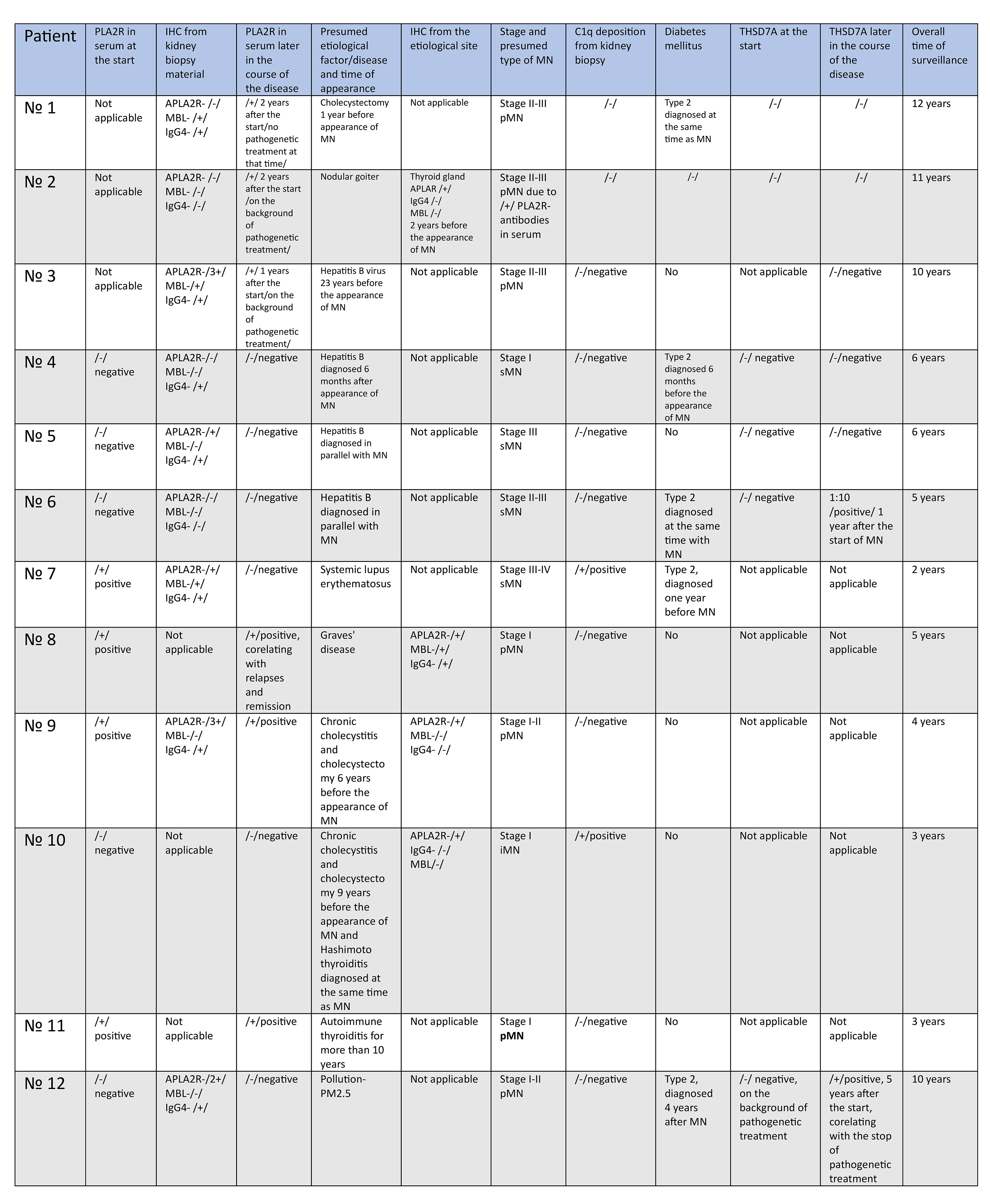

Supplement: Supplementary file 1 [file ijms-26-02206-s001.zip › ijms-3431494-supplementary.png]
